# A Narrative Review of Biologic Therapies for Type 2 Inflammation in Severe Asthma and Eosinophilic Chronic Obstructive Pulmonary Disease (COPD): Mechanisms, Efficacy, and Safety

**DOI:** 10.7759/cureus.110062

**Published:** 2026-06-01

**Authors:** Shan Hemachandra, Jia Li Ngu, Evan N Goutama, Horng Chen Ooi, Angel Angel, Theint Ei Cho, Eva Goh, Arkar M Aung, Ruby S Inayah, Anselm Koo Kai Jie, Nisha Gaur

**Affiliations:** 1 Internal Medicine, Integrity Pulmonary Specialist, LLC, Phoenix, USA; 2 Internal Medicine, Nanjing Medical University, Nanjing, CHN; 3 Pre-Medical Sciences, Basha High School, Chandler, USA

**Keywords:** benralizumab, biologic therapy, dupilumab, interleukin-3 (il3), interleukin-4 (il4), interleukin-5 (il5), mepolizumab, severe asthma, tezepelumab, thymic stromal lymphopoietin (tslp)

## Abstract

Severe asthma affects a minority of patients but accounts for disproportionate morbidity, mortality, and healthcare costs. The development of targeted biologic therapies has revolutionized treatment, offering precision medicine approaches based on underlying inflammatory endotypes. Currently approved biologics target immunoglobulin E (omalizumab), interleukin (IL)-5 or its receptor (mepolizumab, reslizumab, depemokimab, and benralizumab), IL-4 receptor α (dupilumab), and thymic stromal lymphopoietin (tezepelumab). These agents have demonstrated substantial reductions in exacerbation rates, improved lung function, corticosteroid-sparing effects, and enhanced quality of life across diverse patient populations. This review examines the mechanisms of action, clinical efficacy, safety profiles, and optimal patient selection strategies for biologic therapies in severe asthma and eosinophilic COPD.

## Introduction and background

Type 2 (T2) inflammation is a central driver in severe asthma and eosinophilic chronic obstructive pulmonary disease (COPD), mediated by cytokines such as interleukin (IL)‑5, IL‑4, and IL‑13 [1]. IL‑5 promotes eosinophil maturation, survival, and trafficking [2], while IL‑4 and IL‑13 regulate immunoglobulin E (IgE) production, airway hyperresponsiveness, mucus hypersecretion, and epithelial dysfunction [3].

Targeting these pathways with biologics has transformed management of T2‑high phenotypes, reducing exacerbations, improving lung function, and enabling corticosteroid sparing. IL‑5 (mepolizumab, reslizumab, and depemokimab), IL‑5 receptor alpha (IL‑5Rα) inhibitors (benralizumab), IL‑4Rα blockers (dupilumab), IgE blockade (omalizumab), and thymic stromal lymphopoietin (TSLP) inhibition (tezepelumab) represent the current therapeutic armamentarium [4]. Clinical trials have established efficacy, biomarker correlations, and safety, shaping evidence-based use in severe asthma and select eosinophilic COPD populations.

## Review

IL‑5 receptor‑targeted therapy: benralizumab

Benralizumab, a humanized monoclonal antibody directed against IL‑5Rα, induces near‑complete depletion of eosinophils and basophils through antibody‑dependent cellular cytotoxicity [5]. The efficacy of benralizumab for add-on maintenance treatment of severe asthma with an eosinophilic phenotype was established through two landmark randomized, double-blind, parallel-group, placebo-controlled trials: SIROCCO [6] and CALIMA [7]. These pivotal exacerbation trials were conducted over 48 and 56 weeks, respectively, and collectively randomized 2,510 patients (1204 (SIROCCO) and 1306 (CALIMA) aged 12 years and older with severe, uncontrolled asthma. In the phase III SIROCCO and CALIMA trials, benralizumab demonstrated consistent reductions in annualized asthma exacerbation rates (AER) when added to high‑dose inhaled corticosteroids (ICS) and long‑acting β₂‑agonists (LABA) in patients with severe, uncontrolled eosinophilic asthma.

For all exacerbations in patients with eosinophils ≥300 cells/µL, SIROCCO showed that benralizumab reduced exacerbation rates from 1.52 per year (placebo) to 0.74 per year, representing a 51% reduction (rate ratio 0.49, 95% CI 0.37-0.64) [6]. In CALIMA, benralizumab reduced rates from 1.01 per year (placebo) to 0.73 per year, achieving a 28% reduction (rate ratio 0.72, 95% CI 0.54-0.95) [7]. When examining exacerbations requiring emergency department visits or hospitalization, the benefits were substantial in SIROCCO, with benralizumab reducing rates from 0.25 per year (placebo) to 0.09 per year, representing a 63% reduction (rate ratio 0.37, 95% CI 0.20-0.67) [6]. However, in CALIMA, this endpoint showed no significant benefit, with rates of 0.12 per year for benralizumab versus 0.10 per year for placebo (rate ratio 1.23, 95% CI 0.64-2.35) [7]. For the most severe exacerbations requiring hospitalization, SIROCCO demonstrated a 52% reduction with benralizumab (0.07 vs 0.14 per year, rate ratio 0.48, 95% CI 0.22-1.03), though this did not reach statistical significance. CALIMA showed no benefit for this endpoint (rate ratio 1.48, 95% CI 0.65-3.37). These findings indicate that benralizumab's effectiveness in preventing severe exacerbations may vary between studies, with more consistent benefits observed in the SIROCCO trial.

In a post hoc pooled analysis of SIROCCO and CALIMA, patients with baseline blood eosinophils ≥300 cells/µL and fixed airflow obstruction (FAO), postbronchodilator forced expiratory volume in one second/forced vital capacity (FEV₁/FVC) <70%, constituted approximately 63%, while 37% had no FAO [5]. Benralizumab 30 mg administered every eight weeks after three loading doses significantly reduced AER compared with placebo in both subgroups, with a rate ratio of 0.56 (95% CI 0.44-0.71; p < 0.0001) for FAO‑positive patients and 0.58 (95% CI 0.41-0.83; p = 0.003) for FAO‑negative patients. Among patients with baseline blood eosinophil counts ≥300 cells/µL comprising the primary analysis population, benralizumab significantly reduced the annualized asthma exacerbation rate over 48 weeks compared with placebo when administered Q4W (rate ratio 0.55, 95% CI 0.42-0.71; p < 0.0001) or Q8W (rate ratio 0.49, 95% CI 0.37-0.64; p < 0.0001). Both dosing regimens produced significant improvements in prebronchodilator FEV₁ at week 48, with least-squares mean increases of 0.106 L (Q4W) and 0.159 L (Q8W) relative to placebo. Symptom improvement, as measured by the Asthma Control Questionnaire-6 (ACQ-6) [8], reached statistical significance with the Q8W regimen (mean difference −0.25, 95% CI −0.45 to −0.06), but not with Q4W (−0.08, 95% CI −0.27 to 0.12). A subgroup of the CALIMA trial echoed these results: patients with eosinophils ≥300 cells/µL treated with benralizumab experienced a 66% reduction in AER in the Q4W group (rate ratio 0.34, 95% CI 0.11-0.99) and an 83% reduction in the Q8W group (rate ratio 0.17, 95% CI 0.05-0.60), both statistically and clinically significant. Pre‑bronchodilator FEV₁ improved significantly by 0.334 L in the Q4W group (95% CI 0.020-0.647). While symptom scores did not reach significance in this subgroup, safety was comparable to placebo [9].

Analyses stratified by baseline eosinophils ≥150 cells/µL reaffirmed benralizumab’s efficacy: SIROCCO reported a 42% reduction (rate ratio 0.58, 95% CI 0.46-0.74; p < 0.001) while CALIMA showed a 36% reduction (rate ratio 0.64, 95% CI 0.50-0.81; p < 0.001). Improvements in pre‑bronchodilator FEV₁ (0.163 L in SIROCCO, 0.116 L in CALIMA) further substantiated meaningful clinical benefit, although symptom score significance varied by trial [10].

Long‑term extension data from the BORA study indicated durability of effect: crude annual exacerbation rates remained low (~0.56 per year) for Q8W dosing through two years, with sustained lung function and quality‑of‑life benefits and a safety profile consistent with pivotal periods [11]. Biomarker analyses confirmed that increasing eosinophils predicted higher placebo exacerbation rates but not variable benralizumab efficacy across IgE quartiles, indicating eosinophil‑driven mechanisms independent of IgE status.

ZONDA was a randomized, double-blind, parallel-group trial that evaluated benralizumab's oral corticosteroid (OCS)-sparing potential in 220 adult patients with severe asthma requiring daily OCS (7.5-40 mg per day) despite high-dose inhaled therapy [10]. Patients had blood eosinophil counts ≥150 cells/µL and at least one exacerbation in the prior 12 months. Following an eight-week run-in period to optimize OCS dosing, the baseline median OCS dose was 10 mg daily across all treatment groups. Benralizumab achieved a median 75% reduction in daily OCS dose compared to 25% with placebo. Among eligible patients, 52% receiving benralizumab achieved complete OCS elimination compared to 19% with placebo, while 66% achieved ≥50% OCS reduction versus 37% with placebo [10].

**Table 1:** FDA-approved benralizumab dosing and indications Dosing regimens and approved indications are based on patient age, weight, and clinical indication. Data sourced from the FDA prescribing information [12] Q4W: every four weeks; Q8W: every eight weeks

Indication	Population	Weight	Loading Dose	Maintenance Dose
Severe Asthma w/ Eosinophilic phenotype	Adults, Pediatrics ≥12 years	-	30 mg Q4W First 3 doses	30 mg Q8W
Severe Asthma w/ Eosinophilic phenotype	Pediatrics (6-11 years)	<35 kg	10 mg Q4W First 3 doses	10 mg Q8W
Severe Asthma w/ Eosinophilic phenotype	Pediatrics (6-11 years)	≥35 kg	30 mg Q4W First 3 doses	30 mg Q8W
Eosinophilic granulomatosis with polyangiitis (EGPA)	Adults	-	-	30 mg Q4W

Anti‑IL‑5 ligand therapy: mepolizumab

Mepolizumab neutralizes circulating IL‑5, reducing eosinophil activation and survival [13]. The phase III DREAM study demonstrated robust reductions in clinically significant exacerbations across all dosing regimens. Specifically, the 75 mg dose achieved a 48% reduction (rate 1.24 vs 2.40 exacerbations per patient‑year; 95% CI 31-61%; p < 0.0001), with similar magnitudes at higher doses, establishing the biological rationale for IL‑5 inhibition [14].

The MENSA trial reinforced these findings: subcutaneous 100 mg and intravenous mepolizumab at 75 mg significantly reduced AER by approximately 47%(SC)-53%(IV) (p < 0.001) compared with placebo, with improvements in asthma control and quality of life [15]. In the SIRIUS study focusing on oral corticosteroid-dependent patients, mepolizumab recipients achieved a median OCS dose reduction of ~50% versus no reduction in placebo (p = 0.008), reinforcing its steroid‑sparing capabilities [16]*.* The MUSCA trial extended patient‑centered outcomes, showing a mean St. George’s Respiratory Questionnaire (SGRQ) [17] score reduction of −15.96 points vs −7.99 points for placebo (adjusted mean difference −7.97; 95% CI −10.35 to −5.59; p < 0.001) alongside a 31% exacerbation reduction [18].

In severe asthma trials, the difference from placebo in mean change from baseline FEV₁ showed that DREAM (75 mg intravenous) produced changes of 10 mL at Week 12, 5 mL at Week 24, and 61 mL at Week 52; MENSA (100 mg subcutaneous) showed improvements of 52 mL, 76 mL, and 98 mL at Weeks 12, 24, and 32, respectively; and SIRIUS (100 mg subcutaneous) demonstrated changes of 56 mL and 114 mL at Weeks 12 and 24, respectively, with all values accompanied by wide 95% confidence intervals crossing zero [14-16,19].

Extension data from the COLUMBA [20] and COSMOS [21] studies confirmed sustained efficacy and safety. Over 52 weeks in COSMOS, previously treated patients maintained reduced exacerbations and OCS use, while COLUMBA’s ~3.5‑year follow‑up revealed a continued 56% reduction in AER and a ~78% decrease in eosinophil counts, with most adverse events being mild respiratory infections [20,21].

The Nucala (mepolizumab) COPD trials consisted of two randomized, double-blind, placebo-controlled multicenter studies, MATINEE and METREO, which together enrolled 1,640 adults with inadequately controlled COPD and an eosinophilic phenotype. Patients received either Nucala 100 mg or placebo subcutaneously every four weeks, for 52-104 weeks in MATINEE and 52 weeks in METREO, with the efficacy analysis based on 1,266 of the enrolled participants. METREX reported a statistically significant reduction in annualized moderate or severe exacerbations in eosinophilic patients, whereas METREO did not demonstrate significant benefit. The MATINEE trial revisited this concept, reporting an exacerbation rate ratio of 0.79 (95% CI 0.66-0.94; p = 0.011), but limited impact on symptoms and quality of life highlighted the more nuanced role of IL‑5 blockade in COPD relative to asthma [22,23]. 

Real‑world evidence corroborates these findings, with meta‑analyses showing significant reductions in exacerbations (mean difference −2.73 events/year; p < 0.001), FEV₁ gains (~0.18 L; p < 0.001), and decreased OCS use [24]. Table 2 gives the FDA-approved indications for mepolizumab.

**Table 2:** FDA-approved indications for mepolizumab Dosing varies by indication and patient population. Data sourced from the FDA prescribing information [25]. Q4W: every four weeks; CRSwNP: chronic rhinosinusitis with nasal polyps; COPD: chronic obstructive pulmonary disease

Indication	Population	Dosing	Notes
Severe asthma	Adults	100 mg Q4W	-
Severe asthma	Pediatrics (12-17 yrs)	100 mg Q4W	-
Severe asthma	Pediatrics (6-11 yrs)	40 mg Q4W	-
Chronic rhinosinusitis with nasal polyps (CRSwNP)	Adults	100 mg Q4W	Adult patients aged 18 years and older with inadequate response to nasal corticosteroids
CRSwNP	Pediatrics (<18 years)	N/A	-
COPD	Adults	100 mg Q4W	-
Eosinophilic granulomatosis with polyangiitis	Adults	300 mg Q4W at 3 separate sites, at least 2 inches apart	-
Eosinophilic granulomatosis with polyangiitis	Pediatrics (<18 years)	N/A	-
Hypereosinophilic syndrome	Adults	300 mg Q4W at 3 separate sites, at least 2 inches apart	For greater than or equal to 6 months without an identifiable non-hematologic secondary cause.
Hypereosinophilic syndrome	Pediatrics (12-17 yrs)	300 mg Q4W at 3 separate sites, at least 2 inches apart	For greater than or equal to 6 months without an identifiable non-hematologic secondary cause.

Ultra‑long‑acting IL‑5 inhibition: depemokimab 

Depemokimab, an ultra‑long‑acting IL‑5 antagonist with extended half‑life, offers sustained T2 suppression with biannual subcutaneous dosing. The phase III SWIFT‑1 and SWIFT‑2 randomized, double‑blind, placebo‑controlled trials in patients with eosinophilic asthma revealed significant reductions in AER (rate ratio 0.42 [95% CI 0.30-0.59; p < 0.001] in SWIFT‑1 and 0.52 [95% CI 0.36-0.73; p < 0.001] in SWIFT‑2), indicating comparable efficacy to existing IL‑5/IL‑5Rα agents with considerable dosing convenience. Depemokimab also produced improvements in SGRQ total scores (mean difference −2.93; 95% CI −5.48 to −0.38; P = 0.02). In SWIFT-1 and SWIFT-2, the treatment difference versus placebo in pre-bronchodilator FEV₁ for depemokimab was −1 mL (95% CI −89 to 88) and 56 mL (95% CI −43 to 154), for SWIFT-1 and SWIFT-2, respectively [26,27].

Although depemokimab demonstrated efficacy in reducing clinically significant asthma exacerbations, its effects on other important asthma outcomes, including lung function, symptom burden, and corticosteroid requirements, remain uncertain. This may be partially explained by the design and reporting emphasis of the SWIFT-1 and SWIFT-2 trials, in which these secondary endpoints were neither consistently reported nor designated as primary outcomes. In addition, the trials defined eosinophilic asthma using a blood eosinophil threshold of at least 150 cells/µL, which is lower than the 300 cells/µL criterion commonly employed in other IL-5-targeted studies. This broader eligibility criterion may have resulted in the inclusion of patients with less pronounced T2 inflammatory activity, potentially diminishing observable treatment effects beyond exacerbation reduction [26,27].

Consequently, a major limitation is the inability to evaluate treatment effects within clinically relevant subgroups, such as patients stratified by baseline eosinophil counts, oral corticosteroid use, or the presence of comorbid nasal polyposis, owing to the absence of stratified outcome data in the original trials.

Overall, depemokimab shows robust efficacy in reducing exacerbation rates among patients with severe eosinophilic asthma, particularly those at higher risk. Although improvements in health-related quality of life reached statistical significance, these changes did not meet established thresholds for clinical relevance. The FDA has approved depemokimab as a biannual add-on maintenance treatment of severe asthma with eosinophilic phenotype in adult and pediatric patients aged 12 years and older [28].

IL‑4/IL‑13 dual blockade: dupilumab

Dupilumab targets IL‑4Rα, blocking both IL‑4 and IL‑13 signaling and yielding broad suppression of T2 pathways. In the phase III QUEST trial, 1,902 adolescents (aged ≥ 12 years) and adults received dupilumab (200 mg or 300 mg Q2W) or placebo. Dupilumab significantly reduced severe exacerbations across the overall population, with the greatest benefit observed in patients with elevated T2 biomarkers (blood eosinophils ≥300 cells/µL or fractional exhaled nitric oxide (FeNO) ≥25 parts per billion), achieving exacerbation reductions up to 65.8% and rapid, sustained pre‑bronchodilator FEV₁ improvements evident by week 2 [29].

Post hoc analyses reinforced efficacy irrespective of allergic status: among patients with allergic asthma, annualized severe exacerbations decreased by 36.9% (200 mg) and 45.5% (300 mg), with lung function and control benefits comparable in non‑allergic asthma, emphasizing the central role of IL‑4/IL‑13 in both IgE‑mediated and non‑IgE type 2 inflammation [29]. Dupilumab’s effectiveness also extended across neutrophil categories, with significant reductions in exacerbations and FEV₁ improvements regardless of baseline neutrophil count in patients meeting type 2 criteria. In patients with persistent airflow obstruction (post‑bronchodilator FEV₁/FVC <0.70), dupilumab reduced exacerbations by 69% and improved lung function early and durably. Geographic subgroup analyses, including Korean cohorts, confirmed consistent benefits with acceptable safety profiles [30]. 

The VENTURE trial demonstrated meaningful oral corticosteroid-sparing effects: dupilumab reduced mean maintenance OCS dose by 70.1% versus 41.9% with placebo (p < 0.001), alongside a 59% reduction in exacerbations and improved pre‑bronchodilator FEV₁ [31]. Long‑term TRAVERSE data up to 96 weeks showed sustained reductions in OCS use, low annualized exacerbation rates, and maintained lung function and asthma control, without emergent safety concerns [32,33]. 

Beyond asthma, the phase III BOREAS Trial [34,35] and NOTUS Trial [36] extended the therapeutic scope of dupilumab to patients with COPD characterized by T2 inflammation. In BOREAS, a double-blind randomized study involving 939 patients with COPD and blood eosinophil counts ≥300 cells/µL despite background triple inhaled therapy, dupilumab administered at 300 mg every two weeks significantly reduced the annualized rate of moderate or severe exacerbations to 0.78 compared with 1.10 in the placebo group (rate ratio 0.70; 95% CI 0.58-0.86; p < 0.001) [34,35]. Treatment also produced rapid and sustained improvements in lung function, with prebronchodilator FEV₁ increasing by 160 mL at week 12 versus 77 mL with placebo (least squares mean difference 83 mL; p < 0.001), an effect maintained through 52 weeks. Patient-reported outcomes similarly improved: the SGRQ score improved by -9.7 points with dupilumab versus -6.4 with placebo (difference -3.4; p = 0.002), and the Evaluating Respiratory Symptoms in COPD (E-RS-COPD) [37] score improved by -2.7 versus -1.6 (difference −1.1; p = 0.001), respectively, reflecting meaningful reductions in symptom burden and better health-related quality of life. Safety outcomes were comparable between groups, with similar rates of serious adverse events, treatment discontinuations, and deaths. Biomarker analyses further demonstrated that higher baseline eosinophil counts and fractional exhaled nitric oxide (FeNO) predicted greater treatment benefit, supporting a biomarker-guided approach to therapy. The NOTUS trial subsequently reproduced these findings, demonstrating approximately a 34% reduction in exacerbations with associated improvements in FEV₁, thereby reinforcing the clinical relevance of IL-4 and IL-13 pathway inhibition in COPD populations with type 2 inflammatory signatures [36]. 

Table 3 gives the FDA-approved dupilumab dosing and indications.

**Table 3:** FDA-approved dupilumab dosing and indications Loading and maintenance doses vary by indication, age, and body weight. Data sourced from the FDA prescribing information [38]. Q4W: every four weeks; Q2W: every two weeks; CRSwNP: chronic rhinosinusitis with nasal polyps; COPD: chronic obstructive pulmonary disease

Indication	Population	Weight	Loading Dose	Maintenance Dose	Notes
Asthma (moderate–severe/eosinophilic)	Adults and children ≥12 years	-	400 mg or 600 mg	200 mg or 300 mg Q2W	Add-on maintenance
Asthma (OCS-dependent or with AD/CRSwNP)	Adults and children ≥12 years	-	600 mg	300 mg Q2W	Preferred regimen
Asthma	Pediatrics (6–11 years)	15–<30 kg	-	300 mg Q4W	-
Asthma	Pediatrics (6–11 years)	≥30 kg	-	200 mg Q2W	-
Atopic Dermatitis	Adults	-	600 mg	300 mg Q2W	-
Atopic Dermatitis	Pediatrics (6 months–5 years)	5–<15 kg	-	200 mg Q4W	-
Atopic Dermatitis	Pediatrics (6 months–5 years)	15–<30 kg	-	300 mg Q4W	-
Atopic Dermatitis	Pediatrics (≥6 years)	15–<30 kg	600 mg	300 mg Q4W	-
Atopic Dermatitis	Pediatrics (≥6 yrs)	30–<60 kg	400 mg	200 mg Q2W	-
Atopic Dermatitis	Pediatrics (≥6 years)	≥60 kg	600 mg	300 mg Q2W	-
Chronic Spontaneous Urticaria	Adults	-	600 mg	300 mg Q2W	Patients refractory to H1 antihistamines
Chronic Spontaneous Urticaria	Pediatrics (12–17 years)	30–60 kg	400 mg	200 mg Q2W	-
Chronic Spontaneous Urticaria	Pediatrics (≥2 years)	≥60 kg	600 mg	300 mg Q2W	-
COPD	Adults	-	-	300 mg Q2W	Add-on maintenance
CRSwNP	Adults & Pediatrics ≥12 years	-	-	300 mg Q2W	Add-on maintenance
Eosinophilic Esophagitis	≥1 year	15–<30 kg	-	200 mg Q2W	-
Eosinophilic Esophagitis	≥1 year	30–<40 kg	-	300 mg Q2W	-
Eosinophilic Esophagitis	≥1 year	≥40 kg	-	300 mg QW	-
Prurigo Nodularis	Adults	-	600 mg	300 mg Q2W	-
Bullous Pemphigoid	Adults	-	600 mg	300 mg Q2W	Use with tapering oral corticosteroids
Allergic Fungal Rhinosinusitis	Adults	-	600 mg	300 mg Q2W	Must have history of sino-nasal surgery
Allergic Fungal Rhinosinusitis	Pediatrics (≥6 years)	15–<30 kg	-	300 mg Q4W	Must have history of sino-nasal surgery
Allergic Fungal Rhinosinusitis	Pediatrics (≥6 years)	30–<60 kg	-	200 mg Q2W	Must have history of sino-nasal surgery
Allergic Fungal Rhinosinusitis	Pediatrics (≥6 years)	≥60 kg	-	300 mg Q2W	Must have history of sino-nasal surgery

IgE blockade: omalizumab

IgE has long been recognized as central to the pathobiology of allergic asthma. Allergen‑mediated cross‑linking of IgE bound to high-affinity IgE receptor (FcεRI) on mast cells and basophils initiates degranulation and release of histamine, leukotrienes, cytokines, and other mediators that drive bronchoconstriction, airway inflammation, mucus hypersecretion, and hyperresponsiveness. Elevated circulating IgE is closely associated with both disease severity and exacerbation risk in patients with allergic asthma, making IgE an attractive therapeutic target [39]. 

Omalizumab is a recombinant humanized monoclonal antibody that selectively binds the Cε3 domain of free IgE, preventing it from engaging FcεRI on effector cells. This not only reduces circulating free IgE but also downregulates FcεRI expression on mast cells and basophils, attenuating the allergic inflammatory cascade. The clinical efficacy of omalizumab in severe allergic asthma has been established through several pivotal randomized controlled trials and subsequent biomarker analyses [39].

The INNOVATE trial, a multicenter phase III, double‑blind, placebo‑controlled study, enrolled 419 patients with severe persistent allergic asthma inadequately controlled despite high‑dose inhaled corticosteroids and long‑acting β₂‑agonists [39]. Over the 28‑week treatment period, omalizumab significantly reduced the rate of severe asthma exacerbations compared with placebo, with a relative reduction of 26% (rate ratio 0.738, 95% CI 0.552-0.998; p=0.042). Secondary outcomes showed even more pronounced effects: total exacerbation rates were reduced by 44% (p<0.001). Patients treated with omalizumab also experienced clinically meaningful improvements in quality of life, with Asthma Quality of Life Questionnaire (AQLQ) [40] scores increasing by +0.68 points compared with placebo (p<0.001), a change exceeding the minimal clinically important difference and underscoring the therapeutic impact of IgE blockade in this population [39]. 

The larger EXTRA trial extended these findings in a broader cohort of 850 patients aged 12-75 years with severe allergic asthma [41]. Over 48 weeks, omalizumab reduced the mean exacerbation rate to 0.66 events per patient‑year compared with 0.88 in the placebo arm, representing a statistically significant 25% relative reduction (p=0.006). Improvements were observed in health‑related quality of life and symptom control: patients receiving omalizumab had greater increases in AQLQ scores (mean difference 0.29 points; 95% CI 0.15-0.43), reduced daily short‑acting β₂‑agonist use (−0.27 puffs per day), and better symptom scores. Importantly, the overall safety profile was comparable to placebo, with similar rates of adverse events and serious adverse events, affirming omalizumab’s tolerability in routine clinical settings. 

Subsequent biomarker‑based analyses of the EXTRA dataset explored predictors of omalizumab response. Elevated fractional exhaled nitric oxide (FeNO) was associated with a 53% reduction in exacerbation rate with omalizumab versus placebo (95% CI 37-70; p=0.001), whereas patients with lower FeNO showed only a non‑significant 16% reduction. Elevated blood eosinophil counts predicted a significant 32% reduction in exacerbations (95% CI 11-48; p=0.005), while associations with high serum periostin failed to reach conventional significance. These findings suggest that biomarkers of T2 inflammation, particularly FeNO and eosinophils, may enrich the identification of patients most likely to benefit from IgE blockade.

Pooled post hoc analyses have further defined omalizumab’s physiologic effects. In adolescents aged 12-17 with uncontrolled moderate‑to‑severe allergic asthma, omalizumab significantly improved multiple measures of lung function compared with placebo, including percent predicted FEV₁ (+3.0%, 95% CI 0.2-5.7; p=0.035), absolute FEV₁ (+120.9 mL, 95% CI 30.6-211.2; p=0.009), and forced vital capacity (+101.5 mL, 95% CI 8.3-194.6; p=0.033). These physiologic gains were accompanied by significant reductions in circulating eosinophil counts (mean difference −85.9 cells/µL; 95% CI −137.1 to −34.6; p=0.001), demonstrating that omalizumab not only improves airflow but also attenuates inflammatory burden in pediatric populations. Another pooled analysis found that among pediatric patients who experienced exacerbations during therapy, those treated with omalizumab showed significantly greater lung function preservation at week 12 versus placebo, with a 4.11% difference in percent predicted FEV₁ (95% CI 0.93-7.30; p=0.011) and an approximate 80 mL increase in absolute FEV₁ (95% CI 10-140; p=0.017), highlighting the potential of omalizumab to mitigate lung function loss during periods of instability [42]. 

Mechanistic studies have offered further insight. Investigations into T2 innate lymphoid cells (ILC2s) revealed that severe allergic or eosinophilic asthma is associated with ILC2 proliferation and upregulated expression of inflammatory transcription factors and receptors. After six months of biologic therapy (either omalizumab or mepolizumab), reductions in ILC2‑derived cytokines like IL‑5 and IL‑13 were observed, indicating suppression of T2 inflammatory activity. Although mepolizumab appeared to exert more pronounced effects on certain innate inflammatory signaling pathways, both biologics were linked to meaningful clinical improvements in asthma control and corticosteroid reduction [43]. 

Long‑term safety data come from the EXCELS observational cohort, which followed 7,857 patients with moderate‑to‑severe allergic asthma. 21 patients were excluded from the analyses due to prior omalizumab use without active treatment at the time of study enrollment [44]. Among 5,007 individuals treated with omalizumab and 2,829 in a comparison cohort, initial unadjusted analyses showed higher rates of arterial thromboembolic events in the omalizumab group (6.66 vs 4.64 per 1,000 person‑years). After adjustment for baseline severity and comorbidities, this association was not statistically significant (adjusted hazard ratio 1.32, 95% CI 0.91-1.91), suggesting that differences likely reflected underlying cardiovascular/ cerebrovascular risk rather than causal effects of therapy.

Beyond classic allergic asthma, omalizumab has demonstrated clinical effects in non‑atopic phenotypes. A proof‑of‑concept trial in severe non‑atopic asthma showed that omalizumab significantly reduced FcεRI expression on basophils and plasmacytoid dendritic cells (p<0.001) and produced an approximate 250 mL increase in FEV₁ (p=0.032), indicating that IgE‑mediated mechanisms may contribute to disease even in the absence of classical allergen sensitization [45]. Controlled allergen challenge studies further illustrate mechanistic impact: in an environmental exposure chamber using cat allergen, omalizumab reduced the magnitude of FEV₁ decline by approximately 44% versus placebo (p=0.0009), with improved symptom tolerance during exposure [46].

Finally, pooled analyses combining INNOVATE [39], EXTRA [41], and PROSPERO [47] data have shown that omalizumab’s clinical benefits reduced exacerbations and improved lung function. The results remain consistent across demographic and clinical subgroups, including patients with varying comorbidity burdens and across racial categories, with comparable outcomes in Black and White patients. Collectively, these studies confirm that omalizumab provides significant and durable improvements in clinical outcomes across diverse severe asthma populations, anchored mechanistically in IgE suppression and downstream inflammatory modulation.

Table 4 gives the FDA-approved indications for omalizumab.

**Table 4:** FDA-approved indications for omalizumab For asthma, CRSwNP, and IgE-mediated food allergy, dosing is determined by baseline serum total IgE level and body weight using FDA dose determination charts. Dosing for CSU is not dependent on IgE level or body weight. Data sourced from the FDA prescribing information [48]. Q4W: every four weeks; CRSwNP: chronic rhinosinusitis with nasal polyps; CSU: chronic spontaneous urticaria

Indication	Population	Dosage	Notes
Moderate- severe asthma, (+) skin test/ in vitro reactivity to perennial aeroallergen	Adults, Pediatrics ≥6 yrs	75 to 375 mg SC Q2W or Q4W	Inadequately controlled with inhaled corticosteroids.
CRSwNP	Adults	75 to 600 mg SC Q2W or Q4W	Inadequate response to nasal corticosteroids
IgE-Mediated Food Allergy	Adults, Pediatrics ≥1 yrs	75 to 600 mg SC Q2W or Q4W	In conjunction with food allergen avoidance
Chronic Spontaneous Urticaria (CSU)	Adults, Pediatrics ≥12 yrs	150 to 300 mg SC Q4W	Despite H1 antihistamine treatment

Thymic stromal lymphopoietin (TSLP) blockade: tezepelumab

Despite advances in targeted biologics for severe asthma, a significant unmet need persisted for therapies that could address both eosinophilic and non‑eosinophilic disease, including phenotypes with low T2 biomarkers. TSLP is an epithelial‑derived “alarmin” cytokine released in response to airborne irritants, allergens, and viral infections, which sits upstream of multiple inflammatory pathways. Elevated TSLP expression has been associated with greater airway obstruction, increased severity, and relative glucocorticoid resistance, making it a compelling target for broader asthma control [49].

Tezepelumab, a human IgG2λ monoclonal antibody, binds TSLP and inhibits its interaction with the heterodimeric receptor complex, thereby reducing downstream activation of inflammatory pathways implicated across asthma endotypes [49]. Its clinical development program has demonstrated substantial, phenotype‑agnostic efficacy in severe asthma.

The phase III NAVIGATOR trial enrolled 1,061 adolescents and adults (aged 12-80) with severe, uncontrolled asthma despite standard therapy in a randomized, double‑blind, placebo‑controlled design [49]. Tezepelumab significantly reduced annualized asthma exacerbation rates compared with placebo over 52 weeks across the overall population and clinically relevant subgroups, with reductions ranging from approximately 46% to 63% depending on season and exacerbation definitions, all supported by confidence intervals indicating statistical significance. Lung function measures (pre‑ and post‑bronchodilator FEV₁, FVC, FEF₂₅-₇₅, peak expiratory flow) improved significantly versus placebo, with least‑squares mean differences and 95% CIs supporting clinical relevance. Patient‑reported outcomes and biomarker improvements (reduced blood eosinophils and FeNO) were observed early and sustained, evidencing broad therapeutic benefit [49]. 

Post hoc analyses of NAVIGATOR revealed that tezepelumab’s effects on reducing blood eosinophil counts and FeNO were evident as early as week 2 and sustained through 52 weeks, irrespective of perennial aeroallergen sensitization. Serum IgE levels also declined progressively relative to placebo under tezepelumab, indicating suppression of upstream type 2 signaling across allergic and non‑allergic contexts. Among patients meeting eligibility criteria for omalizumab by United States or European Union labeling, tezepelumab reduced exacerbations by 60% (95% CI 44-71) and 68% (95% CI 55-77), respectively, compared with placebo. Importantly, similar reductions were observed in patients not eligible for omalizumab, demonstrating the broader applicability of TSLP inhibition. Patients ineligible for omalizumab, including those without confirmed perennial aeroallergen sensitization, those with serum IgE levels outside the approved dosing range, or those with a non-atopic or T2-low phenotype, may still derive meaningful benefit from tezepelumab, given its mechanism targets an upstream epithelial alarmin independent of IgE status or eosinophilic burden. Particularly marked and statistically significant reductions occurred in subgroups including those with aspirin/non-steroidal anti-inflammatory drug (NSAID) sensitivity, high eosinophil counts, allergen‑triggered exacerbations, and dupilumab‑eligible patients, highlighting the cross‑phenotype efficacy of tezepelumab [50]. 

Exploratory NAVIGATOR analyses focusing on comorbid chronic rhinosinusitis with nasal polyps revealed that tezepelumab reduced annualized exacerbation rates by 85% in patients with nasal polyps and 51% in those without, both with statistically significant confidence intervals. Lung function (FEV₁), Asthma Control Questionnaire-6 (ACQ‑6), and AQLQ improvements were evident in both subgroups, with greater effect sizes in the nasal polyp population. Sino‑Nasal Outcome Test-22 (SNOT‑22) [51] scores were also clinically meaningful in the nasal polyp group, accompanied by significant reductions in type 2 biomarkers (blood eosinophils, FeNO), illustrating tezepelumab’s efficacy in both lower and upper airway disease [52]. 

Seasonal analyses showed that tezepelumab consistently reduced exacerbations year‑round: 63% in winter (95% CI 52-72), 46% in spring (95% CI 26-61), 62% in summer (95% CI 48-73), and 54% in fall (95% CI 41-64). In patients with seasonal allergies, tezepelumab reduced exacerbations during the spring allergy season by 59% (95% CI 29-77) and during the ragweed season by 70% (95% CI 33-87). Perennial allergy subgroups demonstrated consistent reductions across all seasons, reinforcing the broad protective effect of TSLP blockade [53]. 

The DESTINATION trial [54], a multicenter phase III long‑term extension, included 827 patients who continued or switched from parent studies (NAVIGATOR [49]/SOURCE [55]) and assessed outcomes over two years and through a 40‑week follow‑up after treatment cessation. Among 426 patients analyzed in the extended follow‑up, those previously treated with tezepelumab showed gradual shifts in blood eosinophil counts, FeNO, lung function (FEV₁), and asthma control scores toward placebo levels after stopping treatment, yet none returned to baseline, indicating durable clinical benefits beyond active therapy. Total IgE levels also rose later in follow‑up but remained below baseline and placebo, suggesting prolonged partial suppression of allergic inflammation. While formal statistical testing was not emphasized in these extended observations, the trajectory of sustained benefit underscores clinically meaningful persistence of effect [54]. 

OCS‑sparing effects of tezepelumab have also been substantial. The WAYFINDER study, a single‑arm open‑label phase IIIb trial in 382 adults with OCS‑dependent severe asthma (baseline mean OCS 10.8 mg/day), showed that by week 28, 88.9% (95% CI 84.8-92.3) reduced their OCS dose to ≤5 mg/day without loss of asthma control, and 32.2% (95% CI 26.9-37.8) discontinued systemic steroids entirely [56]. By week 52, these proportions were 89.9% (95% CI 85.9-93.1) and 50.3% (95% CI 44.5-56.2), respectively, with serious adverse events in 9.4% of participants. Reductions occurred consistently regardless of baseline eosinophils, FeNO, or allergy status, supporting tezepelumab’s potential for steroid reduction while maintaining disease control. 

Safety profiling in subgroup analyses, including Japanese patients, confirmed that tezepelumab’s tolerability mirrored that of the overall NAVIGATOR population, with similar incidences of common adverse events, such as nasopharyngitis, influenza, upper respiratory tract infection, bronchitis, pharyngitis, and pyrexia, and no reports of treatment‑related anaphylaxis, malignancy, helminth infection, or Guillain-Barré syndrome [57]. 

Collectively, tezepelumab’s clinical program, encompassing NAVIGATOR [49], DESTINATION [54], WAYFINDER [56], and subgroup analyses, demonstrates that upstream inhibition of TSLP yields robust, statistically significant reductions in exacerbation rates, improvements in lung function, biomarker suppression, and OCS sparing across diverse patient phenotypes, including those not traditionally considered T2 high. Its phenotype‑agnostic efficacy reflects the mechanistic breadth of targeting an epithelial alarmin that orchestrates multiple downstream inflammatory pathways.

Table 5 gives the indications and usage of tezepelumab.

**Table 5:** Indications and usage of tezepelumab All indications use a uniform flat dose of 210 mg administered once every four weeks, with no loading dose, weight-based, or indication-specific dose adjustments required. Data sourced from the FDA prescribing information and pivotal clinical trials [58]. Q4W: every four weeks; CRSwNP: chronic rhinosinusitis with nasal polyps

Indication	Population	Dosage
Severe Asthma	Adults, Pediatrics ≥12 yrs	210 mg/1.91 mL Q4W
CRSwNP	Adults, Pediatrics ≥12 yrs	210 mg/1.91 mL Q4W

Intravenous anti-IL-5 therapy: reslizumab

Reslizumab is a humanized monoclonal IgG4 antibody that binds with high affinity to circulating human IL-5, preventing its interaction with the IL-5 receptor and thereby disrupting eosinophil maturation and survival. Unlike other IL-5 targeting agents, reslizumab is administered intravenously at a weight-based dose of 3 mg/kg every four weeks.

The efficacy of reslizumab was demonstrated in two identical phase III trials enrolling adults with inadequately controlled moderate-to-severe asthma and blood eosinophil counts ≥400 cells/µL. In both studies, reslizumab significantly reduced annualized clinical exacerbation rates compared with placebo: in study 1, the rate was reduced from 1.8 to 0.9 events per year (50% reduction, p < 0.0001), and in study 2, from 1.6 to 0.8 events per year (50% reduction, p = 0.0002) [59]. Reslizumab is indicated as an add-on maintenance for patients with severe asthma with an eosinophilic phenotype ≥18 years of age.

Safety profiles

Across all biologics, safety has been favorable and consistent over long-term follow-up. Most adverse events are mild, including nasopharyngitis, upper respiratory tract infections, influenza, bronchitis, and pharyngitis. Serious adverse events are uncommon and comparable to placebo. No new safety signals have emerged in extension studies, and anaphylaxis, malignancy, or helminth infections are rare or absent. Long-term observational data for omalizumab (EXCELS) [44] and extension studies for benralizumab (BORA) [11], mepolizumab (COSMOS/COLUMBA) [20,21], dupilumab (TRAVERSE) [32,33], and tezepelumab (DESTINATION [54], WAYFINDER [56]) support the durability of effect without increased risk, with safety profiles remaining stable across demographic subgroups, comorbidities, and geographic populations. Overall, these agents demonstrate robust tolerability alongside clinical efficacy, supporting their sustained use in severe asthma and T2-high COPD.

Discussion

Across multiple trials, precise targeting of T2 inflammatory pathways consistently improves clinically meaningful outcomes. These agents exemplify a paradigm shift toward precision, biomarker-guided management, enabling sustained disease control, reduced steroid exposure, and improved quality of life in severe asthma and selected COPD populations. Notably, direct head-to-head comparative trials between these biologic agents are currently absent from the literature, representing a significant gap in the evidence base and an important area for future research to guide optimal biologic selection. The recently published 2026 American College of Chest Physicians Clinical Practice Guideline provides a practical framework for biologic selection and switching, recommending that patients who fail to achieve an adequate clinical response after four to six months should be considered for a switch to an alternative agent, with selection guided by OCS dependence, exacerbation frequency, T2 biomarker profile, lung function, and comorbid conditions [60]. The guideline emphasizes that, given the absence of comparative effectiveness trials, biologic therapy can only be individualized through indirect data comparisons, and that shared decision-making remains central to optimizing treatment selection.

From a practical standpoint, OCS dependence serves as an important initial decision anchor. For OCS-dependent patients, anti-IL-5/5Rα agents and dupilumab demonstrate comparable efficacy, though anti-IL-5/5Rα may be preferred in those with markedly elevated eosinophil counts, given the risk of dupilumab-induced hypereosinophilia. Dupilumab is favored over tezepelumab in OCS-dependent patients due to its demonstrated corticosteroid-sparing effect, which tezepelumab has not consistently shown. FeNO represents a clinically useful biomarker for guiding biologic switching; a post-treatment FeNO ≥25 ppb in patients failing anti-IL-5/5Rα therapy supports a switch to dupilumab, as higher FeNO values are associated with substantially greater exacerbation reduction and lung function improvement with this agent. Tezepelumab's upstream mechanism of action affords it unique efficacy across both T2-high and T2-low phenotypes, making it the preferred option for patients who do not meet eosinophilic thresholds. Comorbid conditions, including atopic dermatitis, eosinophilic esophagitis, CRSwNP, and COPD, should also inform biologic selection, as several agents carry additional FDA approvals across these disease domains [60].

Safety profiles across all agents are generally favorable, with serious adverse events uncommon and consistent across long-term extension studies. Nevertheless, several agent-specific considerations merit attention. Omalizumab carries a risk of anaphylaxis, and an unadjusted signal for arterial thromboembolic and cerebrovascular events was identified in the EXCELS cohort [44], though this did not reach statistical significance after adjustment for baseline cardiovascular and cerebrovascular risk, suggesting it reflects underlying patient comorbidity rather than a direct drug effect. Dupilumab requires monitoring of blood eosinophil counts in the first three to six months of therapy, given the risk of transient, typically asymptomatic hypereosinophilia, and rare cases of conjunctivitis and eosinophilic granulomatosis with polyangiitis (EGPA) have been reported. Reslizumab requires infusion center administration with epinephrine availability, given its intravenous route and associated anaphylaxis risk. Helminth infestation monitoring is recommended for all biologics except omalizumab, particularly in patients in endemic regions.

Continued research focusing on comparative effectiveness trials, validated definitions of biologic response and remission, long-term remission outcomes, and cost-effectiveness will be essential to refine treatment algorithms and optimize patient selection in this rapidly evolving therapeutic landscape.

**Figure 1:**
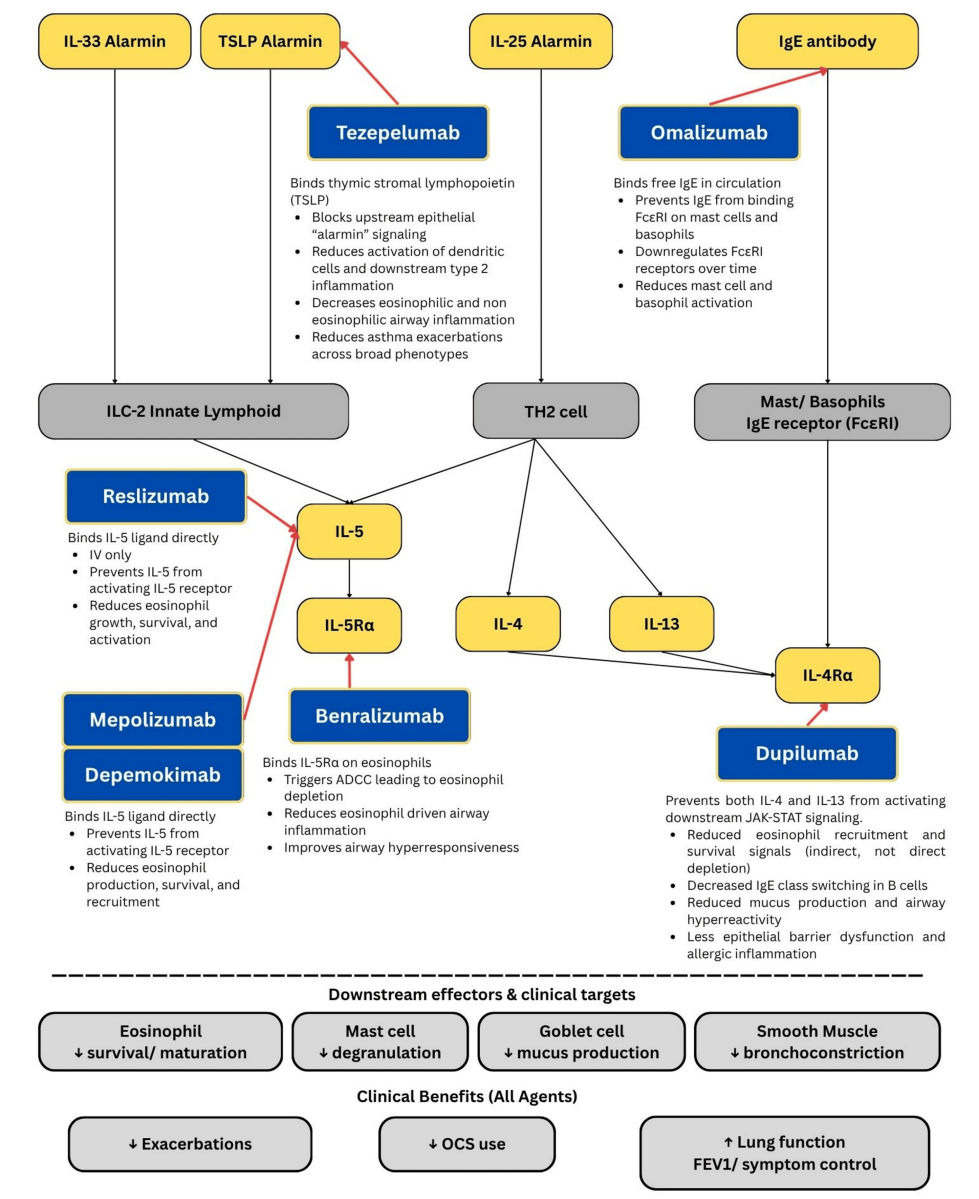
Mechanisms of action of FDA-approved biologic therapies for severe uncontrolled asthma. All agents are used as add-on maintenance therapy to inhaled corticosteroids (ICS) with or without long-acting β₂-agonists (LABA). Dupilumab blocks both IL-4 and IL-13 signaling via the shared IL-4Rα chain. Tezepelumab targets thymic stromal lymphopoietin (TSLP), an upstream epithelial alarmin, conferring efficacy across both type 2 (T2) and non-T2 phenotypes. ICS: inhaled corticosteroids; OCS: oral corticosteroids; LABA: long-acting β₂-agonists; IL: interleukin; ILC-2: innate lymphoid group 2 cells; IgE: immunoglobulin E; FcεRI: high-affinity IgE receptor; TSLP: thymic stromal lymphopoietin; T2: type 2; JAK: Janus Kinase; STAT: Signal Transducer and Activator of Transcription Figure created by authors using Canva (Canva Pty Ltd, Sydney, Australia; www.canva.com).

Figure 1 summarizes the mechanism of action of each of the approved biological agents for the treatment of asthma. Table 6 summarizes pivotal randomized controlled trials evaluating biologic agents in adults with inadequately controlled COPD and an eosinophilic phenotype, including key trial designs, patient populations, dosing regimens, and primary clinical outcomes.

**Table 6:** Type 2 biologic therapies indicated for use in COPD Summary of pivotal randomized controlled trials evaluating biologic agents in adults with inadequately controlled COPD and an eosinophilic phenotype, including key trial designs, patient populations, dosing regimens, and primary clinical outcomes. Data sourced from the FDA prescribing information [12,25,38] and pivotal clinical trials. COPD: chronic obstructive pulmonary disease

Biologic	Target	Key Trials	Population	Dosing / Administration	Main Outcomes
Mepolizumab	IL-5	METREX, METREO [22,23]	COPD patients with eosinophilic phenotype (blood eos ≥150 or ≥300 cells/µL)	Subcutaneous 100 mg Q4W	METREX: 18% reduction in annual moderate/severe exacerbations (rate ratio 0.82; 95% CI 0.68–0.98; p=0.04); METREO: no significant reduction; modest improvements in FEV₁ and SGRQ
Benralizumab	IL-5Rα	GALATHEA, TERRANOVA [61]	COPD patients with eosinophilic phenotype	Subcutaneous 30 mg Q4W or Q8W	No significant reduction in annual exacerbations despite near-complete eosinophil depletion; lung function and quality of life not significantly improved
Dupilumab	IL-4Rα (IL-4/IL-13)	BOREAS [34,35], NOTUS [36]	COPD patients with type 2 inflammation (blood eos ≥300 cells/µL)	Subcutaneous 300 mg Q2W	Reduced annualized moderate/severe exacerbations by 30–34%; improved prebronchodilator FEV₁ (+83 mL at week 12 vs placebo); improved SGRQ (-3.4 points) and E-RS-COPD (-1.1 points)

## Conclusions

Biologic therapies targeting T2 inflammation have fundamentally transformed the management of severe asthma and selected COPD phenotypes, shifting treatment toward precision, mechanism-based therapy. Agents targeting IL-5, IL-5Rα, IL-4/IL-13, IgE, and TSLP consistently demonstrate significant reductions in exacerbation rates, improved lung function, reduced corticosteroid dependence, and enhanced quality of life. Biomarker-guided selection using blood eosinophils, FeNO, and IgE has enabled more personalized treatment strategies, improving outcomes while minimizing exposure to ineffective therapies. Continued research focusing on head-to-head comparisons, long-term remission outcomes, and cost-effectiveness will be essential to refine treatment algorithms and optimize patient selection in this rapidly evolving landscape.
